# SEALIVE: the use of technical vessel-sealing devices for recipient hepatectomy in liver transplantation: study protocol for a randomized controlled trial

**DOI:** 10.1186/s13063-018-2778-1

**Published:** 2018-07-16

**Authors:** Philipp Houben, Elias Khajeh, Ulf Hinz, Phillip Knebel, Markus K. Diener, Arianeb Mehrabi

**Affiliations:** Department of General, Visceral, and Transplant Surgery, University Hospital Heidelberg, Im Neuenheimer Feld 110, 69120 Heidelberg, Germany

**Keywords:** Liver transplantation, Recipient hepatectomy, Surgical technique

## Abstract

**Background:**

The surgical technique used in liver transplantation has undergone constant evolution in an effort to develop a safe, highly standardized procedure. Despite this, the initial step of recipient hepatectomy has not been the focus of clinical research thus far. Due to advanced coagulopathy in liver transplant recipients, this part of the operation still carries the risk of severe hemorrhage. This trial is designed to compare an electrothermal bipolar vessel sealing device (LigaSure™) and an ultrasound dissector (HARMONIC ACE®+7) with standard surgical techniques during the recipients’ hepatectomy in liver transplantation.

**Methods/design:**

In a single-center, prospective, randomized, controlled, parallel, three-armed, confirmatory, open trial, LigaSure™ and HARMONIC ACE®+7 will be compared with standard surgical techniques that use titanium clips and conventional knot-tying ligations during recipient hepatectomy in liver transplantation. Intraoperative total blood loss is the primary endpoint of the trial. Secondary endpoints include blood loss during hepatectomy, the duration of both the hepatectomy and the entire surgical procedure, and blood transfusion requirements of the procedure. To generate reliable data, intraoperative blood loss will be recorded with respect to all rinse fluids during surgery, ascites, and by weighing used swabs. At 80% power and an alpha of 0.025 for both of the experimental groups, 23 subjects will be analyzed per protocol in each study arm in order to detect clinically relevant reduction of intraoperative blood loss. The intention-to-treat analysis will include 69 patients. The follow-up period for each patient will be 90 days for safety reasons, whereas all clinical outcomes will be measured within the first 10 postoperative days.

**Discussion:**

To our knowledge, this is the first prospective, randomized trial comparing two innovative technical methods of vessel sealing and dissection with standard techniques for recipient hepatectomy. This will be done to detect relevant reduction of intraoperative blood loss during liver transplant. The results of the trial are expected to improve patient outcome and safety after liver transplant and to increase the general safety of this procedure.

**Trial registration:**

ClinicalTrials.gov, NCT 03323242. Registered on October 26, 2017.

**Electronic supplementary material:**

The online version of this article (10.1186/s13063-018-2778-1) contains supplementary material, which is available to authorized users.

## Background

Liver transplant (LT) is a well-established procedure for the treatment of end-stage liver disease. Many improvements in the surgical technique have rendered this operation relatively safe. Most important operative innovations after the initial introduction of LT in the clinical routine undertaken by Starzl include the use of venovenous bypass in LT [1, 2]; the piggyback technique with preservation of the recipients’ caval vein [3]; and its modification, which was introduced by Belghiti with side-to-side cavocaval anastomosis [4]. Nevertheless, very few improvements have been introduced in the surgical technique with regard to tissue preparation and sealing the blood vessels during recipient hepatectomy. Due to end-stage liver disease and the recipients’ general and coagulatory condition, hepatectomy carries the risk of severe blood loss (BL), which can impair the outcome after LT. Usually, recipient hepatectomy is carried out as a combination of sharp dissection of the hepatic adhesions to the abdominal wall and the diaphragm and clip or suture ligature of small retrohepatic caval vein branches [5].

With advances in surgical procedures and equipment, modern technologies have been introduced that have shortened operation time and improved surgical outcomes. Exquisite equipment for liver parenchymal transection, such as Cavitron ultrasonic surgical aspirator (Integra LifeSciences, Plainsboro NJ, USA), ultrasonic dissector (USD), LigaSure™ (LS; Covidien, Medtronic, Minneapolis, MN, USA), and TissueLink (Medtronic, Minneapolis, MN, USA), can also be used to reduce hemorrhage in liver resection [6]. The ultrasonic scalpel (Ethicon, Bridgewater, NJ, USA) is a USD that cuts and coagulates tissue using ultrasound at frequencies higher than those used with an ultrasonic aspirator [7]. This device can also serve as a grasper and basically uses a blade that oscillates at 55 kHz, thus producing heat and enabling coagulation of vessels. Recently, its use and potential advantages in open liver resection have been demonstrated. The main technical advancement in this field relates to decreased intraoperative bleeding [8, 9]. Olmez et al. [10] described dissection of the small retrohepatic veins with a USD (HARMONIC™ scalpel; Ethicon) during recipient hepatectomy. Their results showed that this method is safe when compared with conventional knot-tying ligation regarding intra- and postoperative bleeding rate.

The LS electrothermal bipolar vessel-sealing device is another alternative; it applies electrothermal bipolar coagulation and dissection in one step. The LS dissection device seals the tissue first, before it is divided; both tasks are performed with the same device. This may prevent severe bleeding. Furthermore, the sealing device is capable of coping with small liver veins that can be sealed and divided safely without the need for sutures or clips, the latter of which are known for interfering with sufficient “tangential” clamping of the inferior vena cava (IVC) for side-to-side cavocavostomy during piggyback LT [11]. In 2012, Lamattina et al. [12] reported the use of LS devices for recipient hepatectomy in LT. They concluded that LS vessel sealing was an efficient method and that vessel sealing of the caval and portal veins as well as other structures could be safely performed in the setting of end-stage liver disease.

To our knowledge, no randomized clinical trial has been conducted to compare various innovative dissection methods against the standard techniques used for recipient hepatectomy. While LS and USD have been proven to be used safely in several major surgical procedures, including liver resection [13–20], their ability to reduce BL in LT recipient hepatectomy has not yet been evaluated systematically.

## Methods/design

This is a single-center, prospective, randomized, controlled, parallel, three-armed, confirmatory, open trial. This trial is designed to evaluate the potential beneficial effects (superiority design) of using an LS device and USD for recipient hepatectomy during LT. Furthermore, the practicability of these methods is to be investigated. Both the duration of the procedure and the BL during both the entire surgery and the hepatectomy phase of transplant are of interest.

### Hypotheses

Null hypotheses:

• The intraoperative BL does not differ in any clinically relevant extent between the control group and USD with (H0_1_): BL 1 = BL 2.

• The intraoperative BL does not differ in any clinically relevant extent between the control group and LS with (H0_2_): BL 1 = BL 3.

Alternative hypotheses:

• The intraoperative BL differs in a clinically relevant extent between the control group and USD with (H1_1_): BL 1 ≠ BL 2.

• The intraoperative BL differs in a clinically relevant extent between the control group and LS with (H1_2_): BL 1 ≠ BL 3.

### Setting

This investigator-initiated trial is set at a single referral center which is specialized in visceral transplant surgery. All LTs are carried out within a structured, interdisciplinary LT program that has been externally reviewed and accredited.

### Outcome measures

#### Primary endpoint

- Total BL during surgical procedure

#### Secondary endpoints

- BL during recipient hepatectomy
- Time from skin incision to end of hemostasis after hepatectomy
- Time from skin incision to end of skin closure
- Hemodynamic status during surgery: Data on the mean arterial pressure and central venous pressure (CVP) will be obtained at the beginning of hepatectomy after incision and adhesiolysis.
- Coagulation state: International normalized ratio (INR), partial thromboplastin time (PTT), and platelet counts of patients will be recorded pre- and postoperatively until postoperative day (POD) 10.
- Hemoglobin (Hb) level: Hb levels of patients will be recorded pre- and postoperatively until POD 10.
- The number of units of packed red blood cells (PRBC) transfused during surgery
- The number of fresh frozen plasma (FFP) units transfused during surgery
- The number of platelet units transfused during surgery
- Postoperative PRBC and FFP transfusion until POD 10
- Postoperative bleeding: Postoperative hemorrhaging until POD 10 will be recorded and classified as defined by Dindo et al. [21].
- Postoperative morbidity: Postoperative morbidity will be recorded and classified as defined by Dindo et al. [21].
- Retransplant rate
- Conversion rate to alternative methods during recipient hepatectomy in LS and USD groups

### Procedures and methods

#### General description

After incision through the abdominal wall, depending on the randomization result, the electrothermal bipolar vessel sealing device (“LS”, LigaSure™ Maryland Jaw 23 cm Open Instrument; Covidien plc), an ultrasound dissector (“USD”, HARMONIC ACE®+7 Shears; Ethicon), or standard methods applying titanium clips and ligations will be used for mobilization of the liver from its adhesions and ligaments as well as for dissection and coagulation of retrohepatic IVC branches and other small blood vessel-bearing structures. Tissue coagulation and dissection will be carried out in small portions according to the manufacturer’s instructions. The dissection of the small blood vessels and the connective tissue in the hepatoduodenal ligament will also be carried out with LS or USD in the experimental groups, whereas the common bile duct, hepatic artery, and portal vein, as well as the major hepatic veins (left, middle, and right), will be divided with endovascular staplers or conventional knot-tying ligations.

#### Therapeutic effects

Previous studies have reported that both LS and USD devices can be used safely for recipient hepatectomy in LT [10–12]. High-volume LT centers have reported intraoperative BL with average values between 1000 and 2100 ml [22–24]. In our experience, BL during LT could be reduced to about 835 (± 560) ml using LS for recipient hepatectomy [11].

##### Undesirable effects/risks

Participation in the trial will not cause additional invasive treatment (e.g., venous puncture for blood sampling) for the subjects. Like any other surgical procedure, freeing the liver from its adhesions and ligaments and dissecting the retrohepatic IVC branches bears the risk of bleeding and impairs other anatomical structures in the operating field. Nevertheless, preparation and dissection using LS and USD have been proven to reliably control bleeding in daily use during a variety of surgical procedures. A systematic review of randomized controlled trials in which LS was compared with other vessel-sealing devices did not reveal any safety concerns associated with the method [25]. Because LS and USD will be applied according to their designated indication within the approved parameters, no unreasonably hazardous risks are expected.

#### Blinding

Blinding the surgical staff is impossible due to the use of different methods in the three groups under investigation. Because the primary endpoint “total BL” will be precisely recorded during surgery by independent staff and secondary endpoints are objective physiological findings, blinding of the subjects is not needed. Therefore, the open design is not expected to cause any avoidable bias.

#### Randomization protocol

Sixty-nine LT recipients are expected to be randomized into three groups of at least 23 patients each:

- LS group
- USD group
- Control group, using conventional bipolar coagulation devices, surgical suture ligatures, and surgical clips (or any dissecting/coagulating device other than LS)

Randomization will be performed using the “block randomization” method. Each block will contain multiple balanced combinations of the three groups: LS, USD, and control. Blocks will be randomly chosen (using simple randomization software with Excel® [Microsoft, Redmond, WA, USA]), and assignment of the participants to the respective groups will be determined. Randomization will be performed at any time between the inclusion of the patients according to the above-listed inclusion criteria and the beginning of recipient hepatectomy. Randomization will be performed by a member the clinical trial center (Klinisches Studien Zentrum [KSC]) of the surgical department of the University Hospital Heidelberg who is not otherwise involved in the trial. Afterward, the staff will personally inform the responsible surgeon about the treatment group to which the patient has been randomized.

##### Inclusion criteria

- Allocation of donor liver via Eurotransplant (Leiden, The Netherlands) to recipient
- Recipients must be aged 18 years or older
- A signed written informed consent for participation in the trial

##### Exclusion criteria

- High urgency state of recipient
- Previous LT
- Combined organ transplant
- Inability to give informed consent

#### Sample size estimation

The total BL during piggyback LT usually ranges from 1000 to 2100 ml [22–24, 26]. Based on our own preliminary clinical experience on the use of the LS device for recipient hepatectomy, a reduction in BL during LT from 1500 ml to 835 ml with an SD of 560 ml can be expected. Because these results were obtained in an uncontrolled, retrospective study in which all procedures were performed by a single, very experienced surgeon, we have added a “buffer” of 200 ml in favor of a conservative yet robust sample size calculation. Therefore, a reduction in BL from 1500 ml to 1035 ml was chosen for this trial. This effect could be confirmed as statistically significant with a two-sided *t* test at a power of 80% and an alpha of 0.025 as well as a superiority margin of 50 ml with 23 patients in either group. Hence, at least 69 patients in total will be included in the intention-to-treat (ITT) analysis. Differences in the per-protocol and ITT analyses will be of certain interest with respect to the feasibility of the methods under investigation.

#### Course of the study

**Fig. 1:**
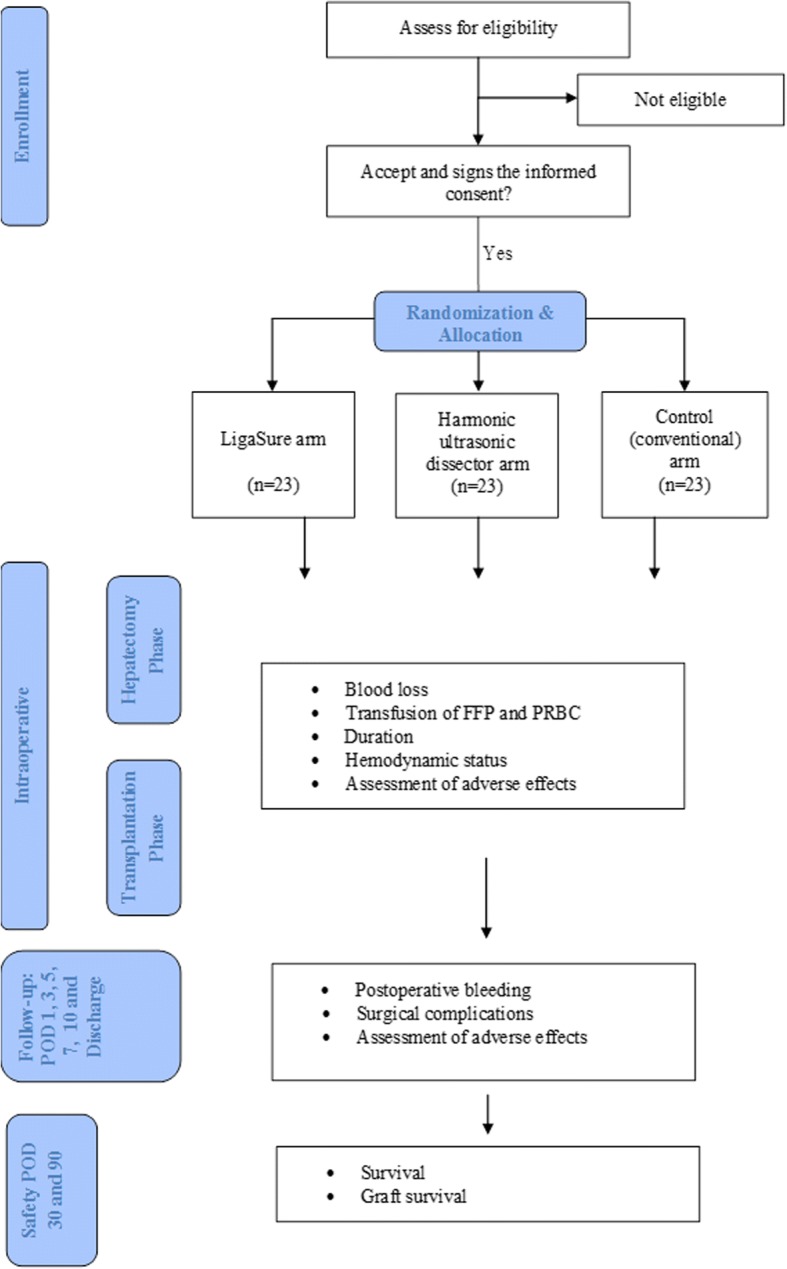
Flowchart of the SEALIVE trial according to Consolidated Standards of Reporting Trials (CONSORT) guidelines

All LT patients who meet our inclusion/exclusion criteria will be informed about the study procedures and their potential benefits. Possible side effects will be clearly declared. Then the patients will be asked to give written consent to participate in the trial via a trial investigator. Baseline clinical and demographic data of the eligible patients will be recorded. Laboratory data will be recorded using routine laboratory tests, which will be performed over the course of the perioperative clinical routine. The patient will be randomized to one of the study arms according to the randomization protocol, and the surgical team will be informed at the time of operation. Figure 1 shows the trial flow diagram.

The entire BL from skin incision to skin closure is defined as “intraoperative BL.” To assess the exact amount of hemorrhage, the volume of fluid in the suction container(s) that are used during the procedure will be recorded in a case report form (CRF) at the end of skin closure procedures. All ascites will be evacuated after opening the abdomen, and the respective amount in milliliters will be documented. Furthermore, the numbers of all small and big surgical swabs will be recorded. All swabs together will be weighed by the end of skin closure procedures along with the drip-catching container in which they are collected. The exact weight in grams will be recorded in the CRF. The exact amount of rinse fluid in milliliters that is used during the procedure will also be recorded in the CRF.

The calculation of total BL will be as follows. The suction container fluid volume (in milliliters) will be added to the weight (in grams) of all surgical swabs at the end of skin closure procedures (A). The difference of the density of the rinse solution (isotonic sodium chloride solution) and blood is approximately 0.055 g/cm^3^. Regarding the accuracy of these measurements, this difference is clinically irrelevant.

The volume of the entire rinse fluid (in milliliters) that is used during the procedure and the amount of ascites (in milliliters) will be added to the known dry weight (in grams) of the respective number of surgical swabs that are used during the procedure and the known dry weight of the drip-catching swab container (B). The total BL is defined as “A” minus “B” in milliliters. Protocol deviations will be recorded.

After the transplant, the routine care protocols (the same as other LT patients) will be performed, and at each visit, the patient will be assessed for BL, transfusion, and postoperative complications. The laboratory tests, including PTT and INR, will be performed in the clinical routine prior to operation as well as 1, 3, 7, and 10 days after operation and at discharge. During the study period, all postoperative surgical complications will be recorded. All study-related data will be handled and stored by the KSC data center. The enrollment period is estimated to be 36 months, with the last patient being enrolled at 39 months.

### Monitoring

Surgical monitoring will be done by intraoperative photographs (to be taken by a member of the KSC) of the procedure being performed according to the randomization result. This will include the situs after opening the abdominal wall, the use of the instrument in the experimental arms, and the ventral surface of the caval vein after removal of the recipient’s liver.

#### Patient time schedule and documentation

##### Visit day − 1

After the inclusion and exclusion criteria are evaluated, if patients are eligible for participation in the study, the following baseline data will be documented:

Demographic characteristics:

- Gender (female/male)
- Age (years)
- Height (cm)
- Weight (kg)

Baseline clinical data:

- Reason for transplant
- Model for End-Stage Liver Disease score
- Baseline laboratory values
- American Society of Anesthesiologists score (according to the anesthesiologist’s protocol)
- Comorbidities (cardiac, pulmonary, renal, autoimmune, hematologic, and infectious; previous abdominal surgery)
- Medication

##### Visit day of surgery

The following data will be recorded intraoperatively according to the study endpoints:

- Duration of hepatectomy
- Duration of whole procedure
- BL during the hepatectomy
- BL during whole procedure
- Transfusion requirements of PRBC and FFP
- Hemodynamic status during hepatectomy and whole procedure (including CVP)
- Presence of portal venous cavernous transformation and/or umbilical varices
- Feasibility of trial methods (rate of conversion to alternative methods during recipient hepatectomy in the LS, USD, and control groups, as well as protocol violations)
- Documentation of the surgeon’s experience in LT (number of cases) and the user’s experience with the applied method (frequent/rare)

##### Visit PODs 1, 3, 5, 7, and 10 and day of discharge

The following data will be recorded postoperatively or taken during the patient’s hospital stay:

- Transfusion requirements
- Hb and platelet count, coagulation parameters
- Surgical complications (i.e., bleeding at site of LS and USD use)
- Serious adverse events (SAE)/treatment-emergent adverse events (TEAE)

##### Visit PODs 30 and 90

**Fig. 2:**
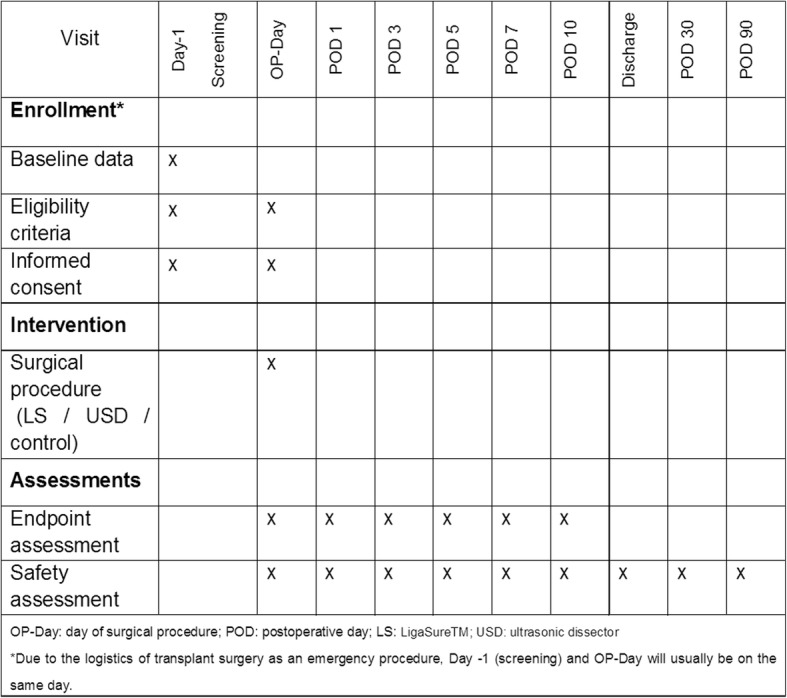
Visit schedule and documentation according to the Standard Protocol Items: Recommendations for Interventional Trials (SPIRIT) guidelines

SAE/TEAE, complications, and mortality will be recorded until POD 90. Figure 2 gives an overview of study visits and documentation of data for each patient.

##### Accompanying therapy

The participant will receive routine care and treatment according to the University Hospital Heidelberg’s manual for LT [27].

##### Safety laboratory

Not applicable.

##### Safety objectives and assessment of safety

To assess the safety of the surgical procedures under investigation, all incidences of TEAEs and SAEs will be documented and evaluated. We will collect only intervention-related events that occur during surgery and follow-up. All TEAEs/SAEs and intervention-related adverse effects will be documented on separate forms and analyzed as part of the safety analysis.

##### SAEs

SAEs are defined as any untoward medical occurrence that meets one or more of the following criteria:

- Results in death
- Is life-threatening
- Requires inpatient hospitalization or prolongation of existing hospitalization
- Results in persistent or significant disability/incapacity
- Requires other intervention(s) to prevent persistent impairment or damage

##### Documentation of SAEs

From the time that the patient approves and signs the informed consent to either the study’s end or the patient’s withdrawal, SAEs must be documented on an SAE form available in the investigator study file. All SAEs that occur from the time between the day of inclusion and discharge must be documented on an SAE form in the CRF. The SAE form will contain the following information: the name of attending physician, a detailed description of the SAE (including event, beginning and duration, severity, outcome, causality to trial intervention, therapy/interventions taken), the consequence for the trial, and a dated signature of the attending physician.

##### Classification of SAEs

- Classification of outcome: The outcome of an SAE at the time of last contact with the patient is classified as follows:
  - Ongoing: Signs and symptoms of the SAE continue.
  - Recovered completely: All signs and symptoms of the SAE have disappeared.
  - Recovered with sequelae: Acute signs and symptoms of the SAE have disappeared, but the sequelae caused by the SAE still exist.
  - Death: The SAE has caused the patient’s death. If a subject has experienced more than one SAE, only the outcome for the SAE directly responsible for death is classified as “death,” whereas the other SAEs are classified according to their specific outcome.
  - Unknown: The outcome is unknown or is implausible, and there is no possibility to complete or verify the information.
- Classification of intensity
  - Mild: a temporary event that is well tolerated by the subject
  - Moderate: an event that results in discomfort for the subject and impairs his/her normal activity
  - Severe: an event that results in substantial impairment of normal activities of subject
- Classification of causality
  - Unrelated: an SAE that does not follow a reasonable temporal sequence from trial treatment and that is likely to have been produced by the subject’s clinical state, other modes of therapy, or other known etiology
  - Possibly related: An SAE that has a reasonable possibility of having been caused by trial treatment. The SAE has a timely relationship to trial treatment(s); however, the event follows no known pattern of response, and an alternative cause seems more likely or there is significant uncertainty about the cause of the event.
  - Definitely related: There is a reasonable possibility that the event may have been caused by trial treatment. A certain event has a strong temporal relationship, and an alternative cause is unlikely.
  - Not assessable: There is insufficient or incomplete evidence to make a clinical judgment regarding the causal relationship to trial treatment.
- Classification of countermeasures: The countermeasures will be documented according to the following rules:
- None: no action taken
- Drug treatment: newly prescribed medication or change in dose of a medication
- Others: other countermeasures (e.g., an operative procedure)

#### Methods of data collection

SAEs will be documented until the end of follow-up of each patient. The results will be evaluated in the clinical results report. Each patient will be asked to report the occurrence of any event to the investigator. At each visit, the investigator will ask the patient if he or she has experienced any of the SAEs noted since the last visit. Each SAE must be reported in the SAE form available in the investigator study file.

#### Reporting of SAEs

SAEs must be reported by the attending physician to the principal investigator within 24 hours after the SAE becomes known. The initial report must be as complete as possible, including details of the current illness, the SAE, and an assessment of the causal relationship between the event and trial treatment.

#### Emergency treatment

During and following the subject’s participation in the trial, the investigator must ensure that adequate medical care is provided. The subject must receive adequate treatment in any clinical situation, including any emergencies and outcomes of the patient that must be checked.

#### Termination criteria

##### Study termination

To assess the trial’s safety, deaths and SAEs will be documented on separate forms and analyzed within the safety analysis. The principal investigator, in consultation with key research associates and the biostatistician, may terminate the trial at any time. Reasons for termination may include high mortality rate as well as incidence or severity of SAEs that indicate a potential health hazard caused by either the study treatment or external evidence which requires premature termination of the trial. Due to the open nature of the trial and the use of fully approved techniques, a data monitoring committee is not necessary in this single-center trial.

#### Patients’ withdrawal

A patient is taken out of the study if he/she withdraws his/her consent to participate at any time without the need to state the reason and without disadvantages for further medical care. The withdrawal of a patient will be documented in the CRF.

#### Statistical design

Dichotomous data and counts will be presented as frequencies. Continuous data will be presented as mean with SD or as median value with a range. Statistical interpretation of the data includes multiple *t* tests with Bonferroni correction for parametric data (including BL) and the Kruskal-Wallis test for nonparametric data. The chi-square test will be used for categorical data. The significance level will be set at alpha ≤ 0.05 (after a Bonferroni adjustment when applicable) representing 95% confidence.

#### Ethical and legal aspects

##### Ethical basics

The study protocol is in accord with the 2013 version of the Helsinki declaration. Participants will voluntarily enroll in the study; consent may be withdrawn at any time, without giving reasons and without disadvantages for further medical care. Execution of the trial is to be carried out by the KSC, an internal division of the Department of General, Visceral, and Transplantation Surgery of the University Hospital Heidelberg. Study personnel will not be additionally compensated for execution of the trial. There is no restricted external financial support for the trial. Participants in the trial will not be compensated financially or otherwise. Because the entire trial will incorporate fully licensed and approved methods in the regular treatment course, no additional compensation for harmful outcomes will be provided.

#### Patient information/informed consent

Patients will be informed in writing and verbally about the nature and scope of the planned study before the start of the study, in particular about the possible benefits to their health and potential risks. Patient approval will be documented by the patient’s signature on the informed consent form. Upon resignation from the study, either data will be destroyed or the patient will be asked whether he/she agrees with further evaluation of the material collected during the trial.

#### Legal basics

##### Vote of the ethics committee

The study protocol will be submitted for review before the trial’s start date by the ethics committee of the University of Heidelberg’s medical faculty. The study will not begin until a written vote from the ethics committee has been received. All relevant changes to the trial protocol will be announced to the ethics committee.

#### Data protection/insight into original patient records

The names of the patients and all other confidential information will be subject to medical confidentiality and the provisions of the German Federal Data Privacy Law (Bundesdatenschutzgesetz [BDSG]) and the European Union General Data Protection Regulation. Transfer of patient data is only possible in pseudonymized form. Third parties will not have access to the original patient records.

## Discussion

This trial will address the important step of recipient hepatectomy during LT because there is little quality data on the implications of this surgical procedure. Of note is the fact that hepatectomy is carried out in patients with end-stage liver disease with normal impaired liver function that leads to coagulopathy, portal hypertension, and thrombocytopenia due to hypersplenism. In this context, finding a portal venous variceal conversion often enhances the already elevated risk of severe intraoperative hemorrhage. Intraoperative BL is associated with inferior outcome after LT, even though the true cause is oftentimes hard to pinpoint [28, 29]. Other than donor organ quality and the condition of the recipient, intraoperative BL is potentially modifiable and therefore deserves special attention to improve the patient’s outcome after LT. The SEALIVE trial is designed to detect and evaluate quantitative reduction in BL. Reducing BL has been proven to be relevant because it has been shown that the average amount of BL in liver resection could be reduced by approximately 400 ml if no transfusion of blood products was necessary [30]. Although intraoperative transfusion requirements of blood products have been shown to correlate with the outcome, they are subject to individual decision-making processes of the anesthesiologists and the center-specific routine [31, 32]. The potential limitation of this unblinded trial design is not believed to incorporate unnecessary bias, because the surgeon cannot be blinded in any case, and the primary endpoint (BL) will be precisely measured intraoperatively by independent staff (Additional file 1).

## Trial status

The latest protocol version of the SEALIVE trial is version 1.6 (June 16, 2018). Recruitment of patients was planned to begin in July 2018.

## Additional file

Additional file 1: SPIRIT 2013 Checklist: Recommended items to address in a clinical trial protocol and related documents. (DOC 122 kb)

## Abbreviations

BL: Blood loss
CRF: Case report form
CVP: Central venous pressure
FFP: Fresh frozen plasma
Hb: Hemoglobin
INR: International normalized ratio
ITT: Intention to treat
IVC: Inferior vena cava
KSC: Clinical trial center (Klinisches Studien Centrum)
LS: LigaSure™
LT: Liver transplant
POD: Postoperative day
PRBC: Packed red blood cells
PTT: Partial thromboplastin time
SAE: Serious adverse event
TEAE: Treatment-emergent adverse events
USD: Ultrasonic dissector
